# Effect of metformin treatment on brain 18F-FDG uptake: a translational study

**DOI:** 10.3389/fradi.2026.1917175

**Published:** 2026-10-01

**Authors:** Rubén Fernández de la Rosa, Gonzalo Cuesta Domingo, Pedro Nespral, Elena Marcos-Macías, Marta Vaillant, Luis García-García, Miguel Ángel Pozo, Jordi A. Matias-Guiu, María Nieves Cabrera-Martin, Mirjam Brackhan, Pablo Bascuñana

**Affiliations:** 1 Cartografía Cerebral, Hospital Clínico San Carlos, IdISSC, Madrid, Spain; 2 Cartografía Cerebral, Instituto Pluridisciplinar, Universidad Complutense de Madrid, Madrid, Spain; 3 BioImaC, Universidad Complutense de Madrid, Madrid, Spain; 4 Nuclear Medicine Department, Hospital Clínico San Carlos, IdISSC, Madrid, Spain; 5 Nuclear Medicine Department, Hospital Puerta de Hierro, Majadahonda, Spain; 6 Departamento de Fisiología, Facultad de Medicina, Universidad Complutense de Madrid, Madrid, Spain; 7 Departamento de Farmacología, Facultad de Farmacia, Universidad Complutense de Madrid, Madrid, Spain; 8 Neurology Department, Hospital Clínico San Carlos, IdISSC, Madrid, Spain

**Keywords:** diabetes, metformin, neurodegeneration, neuroimaging, PET

## Abstract

**Introduction:**

Metformin is one of the most widely used drugs to control diabetes type 2. However, there is no indication of pausing metformin treatment before an ^18^F-FDG PET scan. The aim of this study is to evaluate if metformin, in both patients and mice, can alter brain ^18^F-FDG uptake.

**Methods:**

*In vivo* ^18^F-FDG PET scans were carried out in male mice. Animals were treated with saline or metformin (300 mg/kg) 10 min before ^18^F-FDG or chronically (3 weeks) in drinking water. Separately, patients with non-pathological ^18^F-FDG brain scans were distributed into two groups: metformin-treated (*n* = 12) and control patients (*n* = 27). PET images were structurally normalized to brain templates, corrected to whole-brain activity, and compared using SPM12.

**Results:**

In mice, acute treatment induced a significant hypermetabolism in several brain regions including hippocampus (1.07 ± 0.07 vs 0.94 ± 0.08; *p* = 0.0002) and thalamus (1.17 ± 0.08 vs 1.00 ± 0.08; *p* = 0.0002). Chronic treatment increased tracer uptake only in cortical regions. Similarly, treated patients showed hypermetabolism in the temporal lobe (1.03 ± 0.04 vs 0.97 ± 0.04; *p* < 0.0001) and white matter and lower uptake in frontal cortical regions (1.24 ± 0.04 vs 1.28 ± 0.06; *p* = 0.0165). Linear regression to HbA1c showed a negative correlation to ^18^F-FDG uptake in frontal and parietal cortical subregions, but no correlation to temporal lobe uptake.

**Discussion:**

Our results show that metformin treatment might modify ^18^F-FDG uptake in temporal lobe structures. On the other hand, lower uptake in frontal cortex might be attributable to the diabetic condition. Thus, new studies modifying the treatment regimen in diabetic patients prior to performing an ^18^F-FDG PET are warranted.

## 1 Introduction

Dementia and type 2 diabetes are highly prevalent disorders that often coexist in elderly people (1). Currently, ^18^F-fluorodeoxyglucose positron emission tomography (^18^F-FDG PET) is routinely used for diagnosing dementia-related disorders (2). Dementias are typically associated with brain hypometabolism with distinct spatial patterns depending on the disorder. Alzheimer's disease, the most frequent type of dementia, commonly manifests with hypometabolism in the posterior cingulate gyrus, precuneus, and posterior temporal and parietal lobes whereas frontotemporal dementia, a less common form of dementia, is characterized by hypometabolism in the frontal and anterior temporal lobes and anterior cingulate gyrus (3).

In this context, many patients undergoing ^18^F-FDG-PET evaluation for dementia are also treated with metformin, one of the most widely used drugs to control hyperglycemia in type 2 diabetes (4). Metformin lowers blood glucose levels primarily through inhibition of hepatic glucose production and augmentation of insulin sensitivity (5, 6). In addition, metformin stimulates glucose transporter 4 (GLUT4) translocation in a cyclic adenosine monophosphate-activated kinase (AMPK)-dependent fashion (7). Therefore, metformin can affect tissue glucose uptake, and consequently, ^18^F-FDG uptake. In fact, metformin has been described to stimulate intestinal glucose utilization (8). Various ^18^F-FDG PET imaging studies in metformin-treated diabetic patients and mice showed substantially elevated tracer uptake indicative of increased glucose metabolism in bowel (9–14). Metformin-induced high bowel uptake of ^18^F-FDG crucially impairs PET-based abdominal cancer staging by masking malignant lesions or mimicking pathologic uptake (12). Given that metformin readily crosses the blood-brain barrier (15), treatment with this drug could alter cerebral ^18^F-FDG uptake in a similar fashion and thus, potentially impede a correct diagnosis of dementia spectrum disorders.

However, studies evaluating the effect of metformin treatment on ^18^F-FDG brain distribution are sparse and have delivered inconclusive results. A retrospective clinical ^18^F-FDG PET study in patients with type 2 diabetes demonstrated hypermetabolism in the white matter of the temporal, frontal, and occipital lobes as well as hypometabolism in the parahippocampal gyrus, fusiform gyrus, and ventromedial prefrontal cortex in metformin-treated individuals (16). These authors speculate that this increase in uptake is due to neuroinflammation and glial activation. However, a recent study has shown that metformin reduces neuroinflammation (17), which contradicts this theory. In contrast, a preclinical ^18^F-FDG PET study in mice showed globally reduced tracer uptake in the brain following chronic metformin treatment (18). Thus, additional preclinical and clinical ^18^F-FDG PET studies are necessary to assess whether metformin treatment could potentially confound ^18^F-FDG-based diagnostic brain imaging. Here, we interrogate in both mice and patients how metformin treatment affects the spatial distribution of ^18^F-FDG in the brain.

## 2 Materials and methods

### 2.1 Animals

Adult male C57BL/6J mice (26–29 g; 12–14 weeks of age) were obtained from Charles River Laboratories (Spain). Mice were housed in groups of 5 in individually ventilated cages under controlled temperatures (22 ± 2 °C) and a 12 h light/dark cycle with free access to standard laboratory rodent chow (Safe, France) and tap water. Cardboard houses, nesting material and gnawing bricks (MCViso, Spain) were provided as environmental enrichment. Upon arrival, animals were allowed to adapt to housing conditions and repetitive handling for at least 1 week. Mice were euthanized by decapitation under anesthesia (5% isoflurane in 100% oxygen). All animal experiments were conducted in accordance with the research regulations of the European Union (Directive 2010/63/EU) and Spain (RD53/2013) and with the approval of the local authority (Comunidad de Madrid). All efforts were made to minimize pain, suffering, and the number of animals. The principles outlined in the ARRIVE guidelines and the 3R concept were considered when planning experiments and reporting data.

### 2.2 Experimental metformin treatment

Mice were treated with metformin (reference number 317240, Sigma-Aldrich, Germany) according to two paradigms, acute treatment, employing a longitudinal workflow with each animal serving as its own control, or chronic treatment, comparing metformin-treated and sham-treated groups, to capture complementary experimental conditions. For acute treatment, mice (*n* = 5) received a single intraperitoneal (i.p.) injection of 1) saline before a baseline ^18^F-FDG PET scan and 2) metformin (300 mg/kg in saline) before a second ^18^F-FDG PET scan one week after the baseline scan. For chronic treatment, mice (*n* = 5) received metformin dissolved in drinking water at a dose of 300 mg/kg/day for 3 weeks. Control mice (*n* = 5) received drinking water without metformin. Water consumption rate was controlled 3 times per week to adjust the metformin concentration in water. Chronic treatment was administered through drinking water instead of daily per oral doses to replicate the pharmacokinetic variability inherent to clinical practice.

### 2.3 Preclinical ^18^F-FDG PET imaging procedure and image analysis

Mice receiving acute metformin treatment underwent two ^18^F-FDG PET scans, 1) at baseline after saline injection and 2) one week later after metformin injection. Mice receiving chronic metformin treatment and control mice were subjected to ^18^F-FDG PET after 3 weeks of treatment. Mice were injected i.p. with ^18^F-FDG (5–6 MBq in 0.2 mL saline, Curium Pharma, Spain). Blood glucose levels were measured prior to tracer injection and did not differ between conditions or groups (for baseline vs acute metformin treatment, 200.8 ± 27.4 vs 180.4 ± 43.3 mg/dL; *p* = 0.45; for controls vs chronic metformin treatment, 201.2 ± 13.8 vs 194.6 ± 14.2 mg/dL; *p* = 0.52). For the acute metformin treatment paradigm, vehicle or metformin was administered 10 min before ^18^F-FDG injection. After 45 min of tracer awake uptake, a static 20-minute PET scan followed by a 10-minute computed tomography (CT) acquisition was performed under isoflurane anesthesia (2% in oxygen) in a dedicated small animal PET/CT scanner (Albira ARSII, Bruker, Spain). Images were reconstructed using the maximum likelihood expectation maximization (MLEM) algorithm with corrections for scatter, random, and decay for PET images and the filtered back projection (FBP) algorithm for CT images. PET and CT images were fused automatically by the scanner's reconstruction software (Albira Suite 5.8).

PET images were coregistered to a magnetic resonance imaging (MRI) template (19) using PMOD 4.1 software (PMOD Technologies, Switzerland). To this aim, CT images were coregistered to the MRI template and subsequently mapped to their corresponding PET images previously corrected by SUV calculation. A region of interest (ROI) atlas based on the MRI template was applied to the coregistered images and ^18^F-FDG uptake ratios of ROIs to whole brain were calculated (Supplementary Figure 1A). Additionally, parametric images were obtained by statistical parametric comparisons (SPM12, University College London). For voxel-wise comparison, images were compared at group level by t-test (paired for acute treatment, two-sample for chronic treatment) using SPM12. A significance level threshold of 0.05 without correction for multiple comparisons and a minimum cluster size of 10 voxels were selected. Parametric t maps resulting from each comparison were imported into PMOD and the threshold was set to the corresponding t value. Voxels with a *p* value below 0.05 were anatomically located by coregistration to the MRI template.

### 2.4 *In vitro* autoradiography

Translocator protein (TSPO) expression was assessed using ^3^H-PK11195 (Perkin Elmer, Rodgau, Germany) autoradiography. Sections were dried and preincubated with 50 mM Tris-HCl (pH 7.4, 15 min). Then, samples were incubated with 1 nM ^3^H-PK11195 for 60 min. After ice-cold distilled water washes (2 × 5 min), sections were dried and exposed to autoradiographic film (Blu-Lite UHC™ Western Blotting Film, MTC Bio, USA) for 6 weeks. Autoradiography images were digitalized with a Leica DFC425 camera coupled to a Leica MZ6 stereomicroscope. Autoradiography slices were analyzed using Pmod 4.1. *In vitro* uptake in cortex was correlated to ^18^F-FDG uptake in SPM. The resultant significant cluster was quantified to calculate r.

### 2.5 Patients

^18^F-FDG images from thirty-nine patients with no pathological diagnosis were retrospectively analyzed. The protocol was approved by the Ethics Committee with the 1964 Helsinki declaration and its later amendments. Written informed consent was obtained from all individual participants. Patients were divided into two groups: control (no antidiabetic treatment; *n* = 27) and metformin-treated patients (diabetes type II patients; *n* = 12). Seven patients were taking additional anti-diabetic treatment (3 with additional insulin treatment). Groups did not differ in age or sex distribution (Table 1). Metformin-treated patients had elevated glycosylated hemoglobin in comparison to control subjects (7.07 ± 1.15 vs 5.77 ± 0.36, *p* < 0.001).

**Table 1:** Patient characteristics. Groups were similar in age and sex proportion. Diabetic patients showed elevated glycosylated hemoglobin (HbA1c) values.

Characteristic	Control	Metformin
N	27	12
Gender (% females)	44.4	35.7
Age	67 ± 9.5	69 ± 11.5
Glucosylated Hgb	5.77 ± 0.36#	7.07 ± 1.15***

N, group size. #HbA1c data was not available for 5 control patients. ***p < 0.001.

### 2.6 Clinical ^18^F-FDG PET imaging procedure and image analysis

PET/CT imaging was performed using a Siemens Biograph True Point Platform. Patients fasted for a minimum of 6 h before receiving a dose of ^18^F-FDG (≈185 MBq/10 mL saline i.v.). A static PET image was acquired through a sinogram bed 30 min after administration of the tracer. CT parameters were set as: 130/40/1 (kVp/effective mAs/rotation time); slice thickness of 3 mm; reconstruction interval of 1.5 mm; and pitch of 0.75. Subsequently, iterative 3D image reconstruction was performed using the True X method with two iterations and 21 subsets.

Reconstructed images were pre-processed and analyzed using Pmod 4.1 (Pmod software, Bruker, Germany). First, images were corrected by SUV calculation and coregistered to an in-house ^18^F-FDG PET template. Then, brain uptake was normalized to whole-brain uptake, a ROI map was applied and the SUVR of 65 brain regions quantified (Supplementary Figure 1B). In addition, normalized SUVR images were used for statistical parametric comparison using SPM12 (The Wellcome Trust Centre for Neuroimaging, UCL, UK). In addition, correlation between ^18^F-FDG uptake and glycosylated hemoglobin (HbA1c) in metformin-treated patients was calculated using the linear regression model in SPM12. Resulting parametric images were fused to the ^18^F-FDG PET template in Pmod 4.1 and the threshold set to show only significant voxels (*p* < 0.05 uncorrected).

### 2.7 Statistics

All data are presented as mean ± standard deviation. Experimental group sizes were calculated as independent groups with an *α* of 0.05 and a power of 80%, resulting in a minimum group size of 4 for mice and 8 for patients. Statistical analyses were performed using Prism10 (GraphPad Software, USA). Paired Student's t test was used to compare ^18^F-FDG uptake at baseline and after acute metformin treatment in mice. Unpaired Student's t test was used to compare ^18^F-FDG uptake between mice receiving chronic metformin treatment and control mice and between metformin-treated patients and control subjects. Correlation studies were performed using Pearson's correlation test. Values of *p* < 0.05 were considered statistically significant.

## 3 Results

### 3.1 Metformin elevates brain ^18^F-FDG uptake in a region-specific manner in mice with a greater effect when applied acutely

To non-invasively evaluate the effect of a single dose of metformin on cerebral glucose metabolism, we performed longitudinal ^18^F-FDG PET imaging in mice. We observed increased ^18^F-FDG uptake in the hippocampus (1.07 ± 0.07 vs 0.94 ± 0.08; mean difference 0.13, 95% CI: 0.11–0.16; *p* = 0.0002; *η*^2^ = 0.98), thalamus (1.17 ± 0.08 vs 1.00 ± 0.08; mean difference 0.17, 95%CI: 0.14–0.21; *p* = 0.0002; *η*^2^ = 0.98), midbrain (1.15 ± 0.08 vs 0.98 ± 0.09; mean difference 0.18, 95%CI: 0.11–0.24; *p* = 0.0018; *η*^2^ = 0.93), and striatum (1.23 ± 0.03 vs 1.14 ± 0.07; mean difference 0.09, 95%CI: 0.02–0.16; *p* = 0.0248; *η*^2^ = 0.75) after acute metformin treatment compared to baseline conditions (Figures 1A,B). Statistical parametric mapping (SPM) analysis confirmed this spatial pattern of elevated ^18^F-FDG uptake after a single metformin dose, showing clusters of activated voxels in hippocampus, thalamus, and subregions of cortex and striatum (Figure 1C).

**Figure 1:**
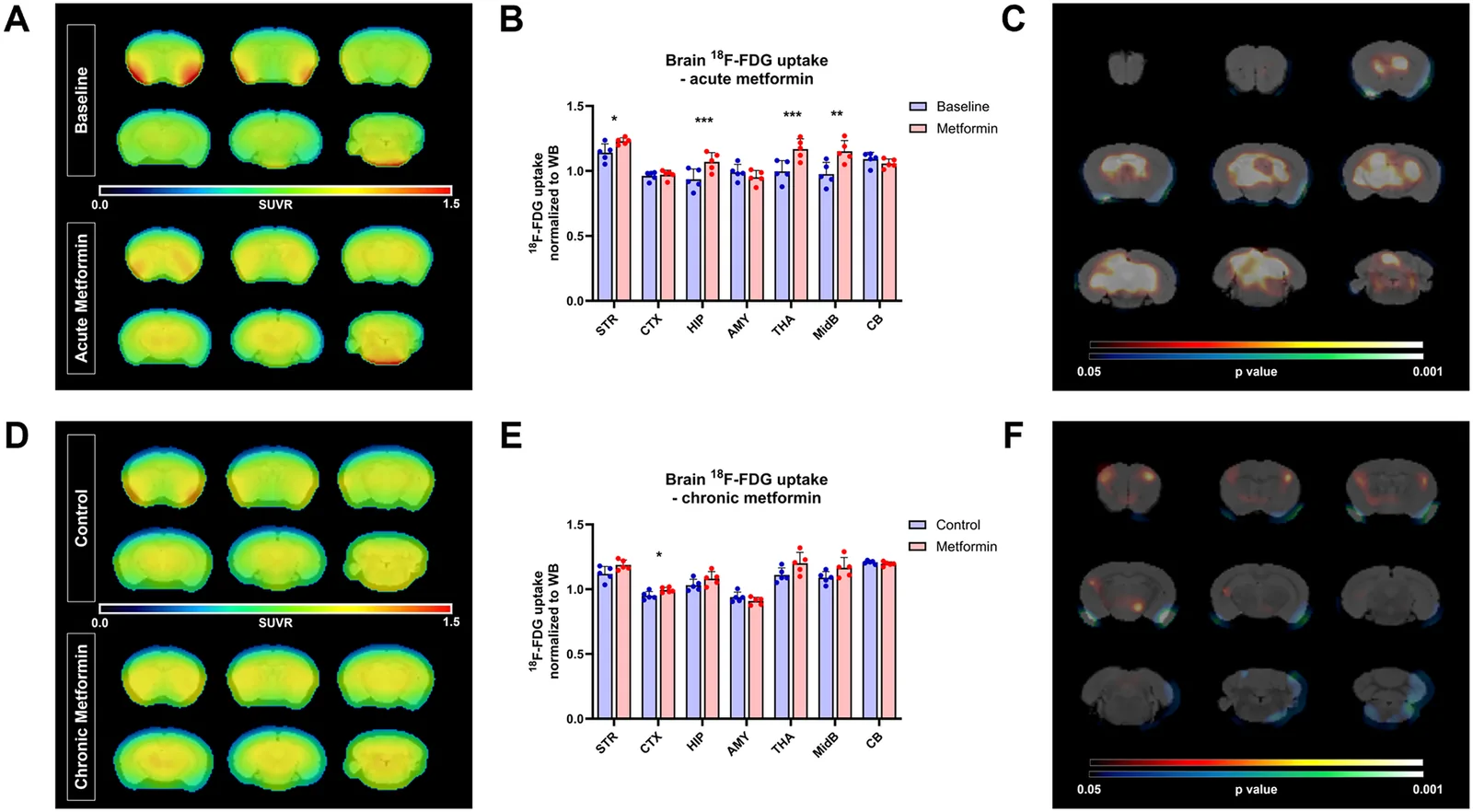
Metformin elevates brain ^18^F-FDG uptake in a region-specific manner in mice with a greater effect when applied acutely. **(A)** Average brain images of ^18^F-FDG SUVR (normalization to WB) in mice at baseline (upper panel) and after acute metformin treatment (lower panel). **(B)** Quantification of ^18^F-FDG uptake normalized to WB activity in brain regions of interest in mice (*n* = 5) at baseline (blue) and after acute metformin treatment (red). Paired two-tailed t-test *p < 0.05 (*p* = 0.0248 in STR), **p < 0.01 (*p* = 0.0018 in MidB), ***p < 0.001 (*p* = 0.0002 in HIP, *p* = 0.0002 in THA). **(C)** Parametric t-maps resulting from comparing normalized ^18^F-FDG uptake between baseline and after acute metformin treatment (paired t-test). **(D)** Average brain images of ^18^F-FDG SUVR (normalization to WB) in control mice (upper panel) and mice receiving chronic metformin treatment (lower panel). **(E)** Quantification of ^18^F-FDG uptake normalized to WB activity in brain regions of interest in control mice (*n* = 5, blue) and mice subjected to chronic metformin treatment (*n* = 5, red). Unpaired two-tailed t-test *p < 0.05 (*p* = 0.0328 in CTX). **(F)** Parametric t-maps resulting from comparing normalized ^18^F-FDG uptake between control mice and mice receiving chronic metformin treatment (unpaired t test). In **(B)** and **(E)**, data are shown as mea*n* ± SD. In **(C)** and **(F)**, clusters of voxels with *p* < 0.05 are shown (minimum cluster size of 10 voxels). SUVR, standardized uptake value ratio; WB, whole brain; STR, striatum; CTX, cortex; HIP, hippocampus; AMY, amygdala; THA, thalamus; MidB, midbrain; CB, cerebellum.

Next, we investigated the effect of chronic metformin treatment on the cerebral ^18^F-FDG PET signal in mice to create an experimental setting closer to clinical conditions. ^18^F-FDG PET in mice receiving chronic metformin treatment identified modestly elevated tracer uptake that was limited to the cortex, compared to control mice (0.99 ± 0.02 vs 0.95 ± 0.03; mean difference 0.04, 95%CI: 0.005–0.082; *p* = 0.0328; *η*^2^ = 0.34) (Figures 1D,E). Accordingly, SPM analysis revealed clusters of increased ^18^F-FDG uptake mainly in rostral areas of the cortex corresponding to the motor and somatosensory cortices, and to a lesser extent in the thalamus and hippocampus (Figure 1F). Additionally, we found clusters of decreased ^18^F-FDG uptake in the amygdala and cerebellum, which are most likely attributable to spillovers of the radiotracer from surrounding tissues.

In addition, we performed ^3^H-PK11195 autoradiography to evaluate translocator protein (TSPO) expression for the chronic treatment paradigm. Autoradiography showed a non-significant increase in cortical TSPO expression in chronically treated animals compared to age-matched controls (36.7 ± 2.2 vs 39.0 ± 1.7; mean difference 2.3, 95%CI: −0.53–5.16; *p* = 0.098 (Figure 2A)). Interestingly, SPM linear regression showed a positive correlation between cortical TSPO expression and ^18^F-FDG uptake in a cluster placed in frontal cortex (Figure 2B). This ROI was quantified to perform Pearson's correlation test confirming the correlation found with SPM [r = 0.73, *p* = 0.017 (Figure 2C)].

**Figure 2:**
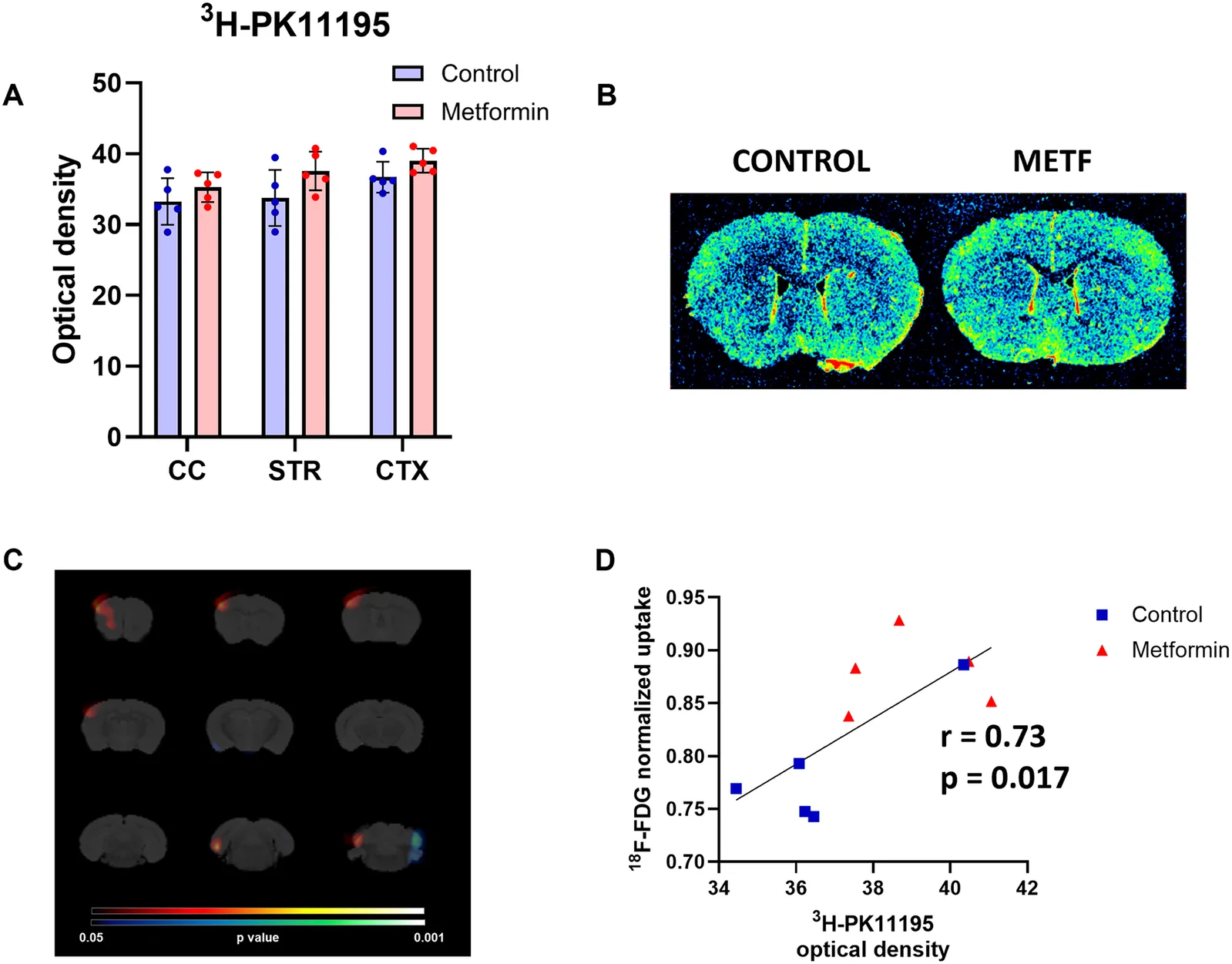
TSPO expression correlates to ^18^F-FDG uptake in mouse cortex. **(A)** Bar graph showing quantification of ^3^H-PK11195 binding in mouse brain slices. Autoradiography shows a non-significant increase in metformin-treated animals compared to their controls. **(B)** Representative ^3^H-PK11195 autoradiography slices for each experimental group. **(C)** SPM regression analysis shows significant (*p* < 0.05) correlation between ^3^H-PK11195 cortical binding and ^18^F-FDG uptake in a cluster of voxels located in frontal cortex. **(D)** Pearson's correlation test shows a significant correlation between TSPO expression and ^18^F-FDG uptake.

### 3.2 Metformin treatment in patients alters ^18^F-FDG uptake in the frontal and temporal lobes

To assess the effect of chronic metformin treatment on cerebral ^18^F-FDG uptake in patients, we retrospectively analyzed ^18^F-FDG images from control and metformin-treated subjects without pathological brain activity. Average brain SUVR images appeared similar between control and metformin-treated patients (Figure 3A). However, quantification of SUVR in ROIs of metformin-treated patients compared to control subjects (Table 2; Supplementary Table 1) revealed decreased ^18^F-FDG uptake in the superior frontal gyrus (1.24 ± 0.04 vs 1.28 ± 0.06; mean difference −0.05, 95%CI: −0.09 to −0.01; *p* = 0.0165; *η*^2^ = 0.15), middle frontal gyrus (1.31 ± 0.06 vs 1.35 ± 0.06; mean difference −0.04, 95%CI: −0.09 to −0.002; *p* = 0.0405; *η*^2^ = 0.11), and inferior frontal gyrus (1.27 ± 0.04 vs 1.30 ± 0.05; mean difference −0.03, 95%CI: −0.06 to −0.002; *p* = 0.0402; *η*^2^ = 0.11) and elevated ^18^F-FDG uptake in the hippocampus and parahippocampal gyrus (1.03 ± 0.04 vs 0.97 ± 0.04; mean difference 0.07, 95%CI: 0.04–0.10; *p* < 0.0001; *η*^2^ = 0.34) and amygdala (0.94 ± 0.07 vs 0.87 ± 0.07; mean difference 0.08, 95%CI: 0.03–0.12; *p* = 0.0020; *η*^2^ = 0.23) (Figure 3B). We observed similar results with SPM analysis, revealing clusters of decreased ^18^F-FDG uptake in the frontal lobe and increased ^18^F-FDG SUVR in temporal regions (Figure 3C). In addition, SPM images showed elevated ^18^F-FDG uptake in white matter areas that were not analyzed with the ROI approach. When performing linear regression to HbA1c values for each metformin-treated patient, parametric images showed a negative correlation in frontal and parietal cortical subregions (Figure 3D).

**Figure 3:**
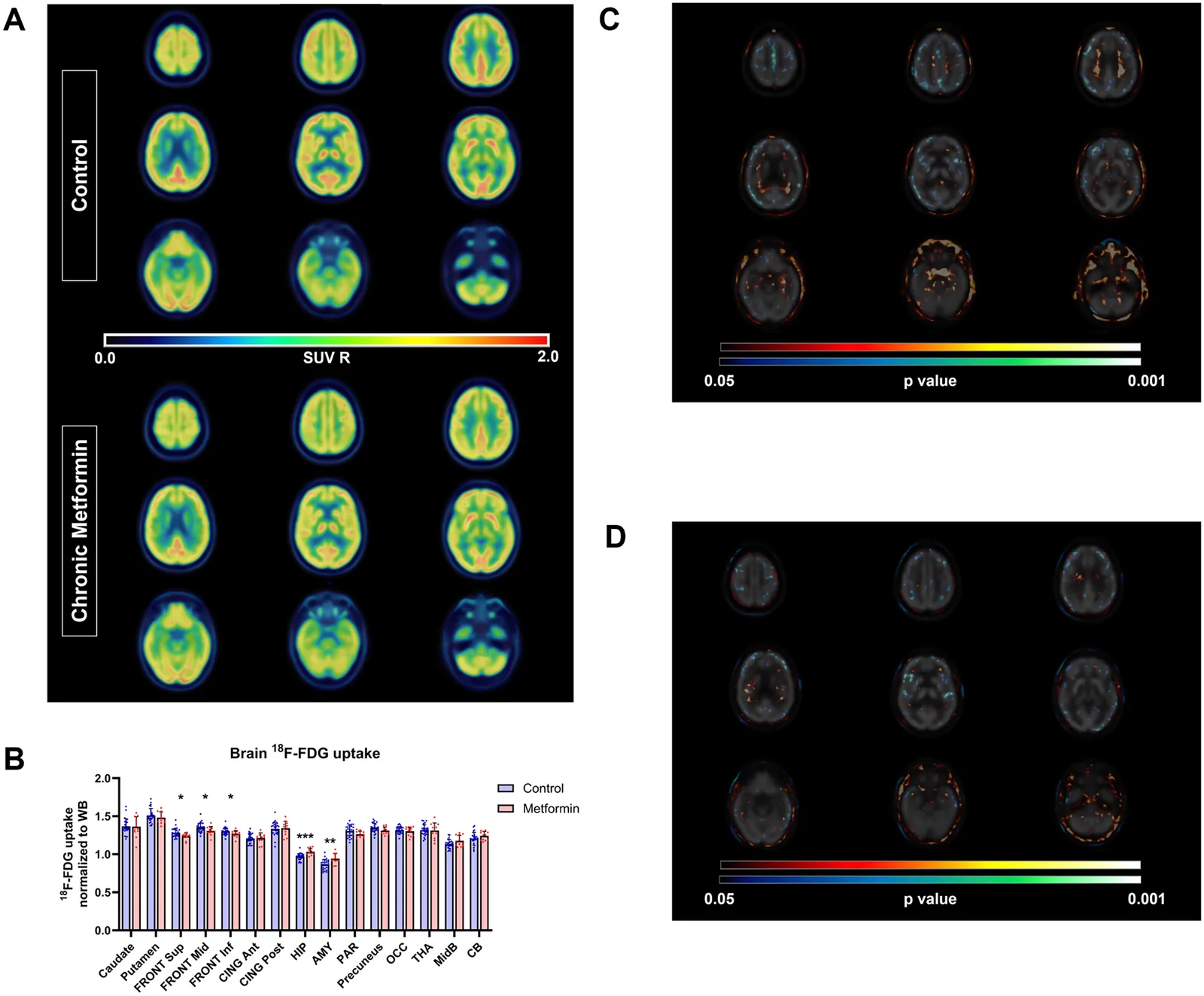
Metformin treatment in patients alters ^18^F-FDG uptake in the frontal and temporal lobes. **(A)** Average brain images of ^18^F-FDG SUVR (normalization to WB) in control subjects (upper panel) and patients receiving chronic metformin treatment (lower panel). **(B)** Quantification of ^18^F-FDG uptake normalized to WB activity in brain regions of interest in control subjects (*n* = 27, blue) and patients under chronic metformin treatment (*n* = 12, red). Unpaired two-tailed t-test *p < 0.05 (*p* = 0.0165 in Frontal Sup, *p* = 0.0405 in Frontal Mid, *p* = 0.0402 in Frontal Inf), **p < 0.01 (*p* = 0.002 in AMY), ***p < 0.001 (*p* < 0.0001 in HIP). Data are shown as mean ± SD. **(C)** Parametric t-maps resulting from comparing normalized ^18^F-FDG uptake between control subjects and patients receiving chronic metformin treatment (unpaired t test). Clusters of voxels with *p* < 0.05 are shown (minimum cluster size of 100 voxels). **(D)** SPM regression analysis of HbA1c values and ^18^F-FDG uptake reveals a significant negative correlation in frontal and parietal cortical subregions. SUVR, standardized uptake value ratio; WB, whole brain; FRONT Sup, superior frontal gyrus; FRONT Mid, middle frontal gyrus; FRONT Inf, inferior frontal gyrus; CING Ant, anterior cingulate cortex; CING Post, posterior cingulate cortex; HIP, hippocampus and parahippocampal gyrus; AMY, amygdala; PAR, parietal lobe; OCC, occipital lobe; THA, thalamus; MidB, midbrain; CB, cerebellum.

**Table 2:** ^18^F-FDG uptake quantification in control and metformin-treated patients. Table shows ROI SUVR value shown as mean ± SD in statistically significant brain regions. Right column shows *p* value of the t-test comparison between both groups.

Region	Control	Metformin	*p*-value
Left Precentral Gyrus	1.326 ± 0.049	1.29 ± 0.084	0.125
Right Precentral Gyrus	1.305 ± 0.054	1.257 ± 0.068	**0** **.** **032**
Left Supplementary Motor Area	1.37 ± 0.052	1.302 ± 0.058	**0**.**001**
Right Supplementary Motor Area	1.382 ± 0.067	1.278 ± 0.077	**0**.**000**
Left Olfactory Cortex	1.114 ± 0.054	1.158 ± 0.05	**0**.**025**
Right Olfactory Cortex	1.111 ± 0.048	1.134 ± 0.077	0.286
Left Frontal Superior Gyrus	1.293 ± 0.062	1.243 ± 0.044	**0**.**019**
Right Frontal Superior Gyrus	1.29 ± 0.059	1.231 ± 0.043	**0**.**004**
Left Frontal Middle Gyrus	1.377 ± 0.064	1.33 ± 0.057	**0**.**043**
Right Frontal Middle Gyrus	1.342 ± 0.061	1.286 ± 0.059	**0**.**015**
Left Frontal Inferior Gyrus	1.312 ± 0.056	1.284 ± 0.043	0.137
Right Frontal Inferior Gyrus	1.297 ± 0.045	1.254 ± 0.047	**0**.**012**
Left Hippocampus Parahippocampus	0.969 ± 0.038	1.048 ± 0.051	**<0**.**001**
Right Hippocampus Parahippocampus	0.955 ± 0.042	1.019 ± 0.04	**<0**.**001**
Left Amygdala	0.855 ± 0.059	0.946 ± 0.064	**<0**.**001**
Right Amygdala	0.856 ± 0.063	0.94 ± 0.072	**0**.**001**
Left Calcarine Sulcus	1.472 ± 0.066	1.442 ± 0.062	0.211
Right Calcarine Sulcus	1.45 ± 0.069	1.392 ± 0.087	**0**.**039**
Left Cuneus	1.513 ± 0.068	1.479 ± 0.058	0.155
Right Cuneus	1.506 ± 0.058	1.438 ± 0.072	**0**.**005**
Left Fusiform Gyrus	1.229 ± 0.05	1.29 ± 0.039	**0**.**001**
Right Fusiform Gyrus	1.209 ± 0.055	1.238 ± 0.053	0.147
Left Postcentral Gyrus	1.272 ± 0.051	1.243 ± 0.071	0.165
Right Postcentral Gyrus	1.284 ± 0.051	1.244 ± 0.058	**0**.**042**
Left Angular Gyrus	1.324 ± 0.084	1.275 ± 0.064	0.090
Right Angular Gyrus	1.366 ± 0.075	1.281 ± 0.07	**0**.**003**
Left Precuneus	1.35 ± 0.053	1.301 ± 0.04	**0**.**009**
Right Precuneus	1.368 ± 0.062	1.321 ± 0.061	**0**.**038**
Left Paracentral Lobule	1.348 ± 0.085	1.285 ± 0.069	**0**.**037**
Right Paracentral Lobule	1.365 ± 0.088	1.286 ± 0.083	**0**.**017**
Left Parietal Lobe	1.328 ± 0.066	1.276 ± 0.067	**0**.**038**
Right Parietal Lobe	1.309 ± 0.07	1.237 ± 0.076	**0**.**009**

*P*-values below 0.05 (significant) are shown in bold.

## 4 Discussion

Currently, ^18^F-FDG PET is routinely used as a tool to diagnose Alzheimer's disease and other dementia-related disorders as they show distinct uptake patterns (2). However, a large proportion of the target population is on antidiabetic treatment, with metformin being the most common method of treatment. Thus, it is key to investigate whether metformin treatment can alter ^18^F-FDG uptake and confound the diagnosis. Here, we have evaluated the effect of metformin on ^18^F-FDG uptake using naïve mice and, retrospectively, in our patient population. We found ^18^F-FDG uptake alterations in both mice and patients that might confuse the assessment of PET neuroimaging and, therefore, hinder diagnosis.

Metformin is the first-line oral treatment for diabetes mellitus type 2 (20, 21). However, its exact mechanism is still not fully elucidated. It is known that metformin lowers blood glucose by inhibiting gluconeogenesis and increasing sensitivity to insulin^5^. In addition, it can regulate cyclic AMPK production, glucagon receptor signaling, GLUT1 expression and GLUT4 translocation (7, 22). Therefore, metformin not only regulates blood glucose but can also affect glucose uptake, and consequently, ^18^F-FDG uptake.

Acute treatment in mice induced a widespread increase of ^18^F-FDG uptake. This increase might be due to the effect of metformin on the mitochondrial complex I. Mitochondrial complex I inhibition is a well-established acute effect of metformin that shifts cellular energy metabolism toward glycolysis, increasing glucose uptake (23). However, chronic metformin treatment in mice showed only an increased uptake in some subregions of the cortex. Thus, ^18^F-FDG brain uptake might adapt to the effect of metformin after a long treatment as it would be the case of patients. Nevertheless, we cannot rule out that it may reflect differences in pharmacokinetics and adaptation to sustained exposure. Thus, metformin concentration in blood was different in acute and chronic animals at the time of the imaging. While this design was intentional to capture the pharmacokinetic variability inherent to clinical practice, we cannot fully exclude that the route of administration itself, and the resulting difference between a sharp peak plasma concentration and sustained low-level exposure, may have contributed to the distinct spatial patterns observed between experiments. Additionally, we detected increased cortical TSPO expression that correlated to elevated ^18^F-FDG uptake in chronically treated mice. This increase might be indicative of altered mitochondrial function. In fact, chronic metformin treatment in mice and rats has recently been shown to increase the mitochondrial area in neuronal axons and glial cytoplasm and to be associated with increased mitochondrial metabolism transcripts (24). Thus, elevated ^18^F-FDG uptake observed after metformin treatment in our study could be in part due to mitochondrial alterations.

In contrast to our results, a previous study showed generalized hypometabolism in chronically treated mice^18^. However, there are several methodological differences to our study. First, the authors administered a metformin dose (750 mg/kg/day) of more than twice the dose we administered (300 mg/kg/day). While metformin could alter ^18^F-FDG brain uptake in a dose-dependent manner, we would not expect a reversal of the effect when doubling the dose. Second, the authors used BALB/c mice whereas we used C57BL/6J mice. Indeed, the two strains show brain-regional differences in ^18^F-FDG brain uptake *per se* (25), which could have contributed to discrepancies. Finally, the authors subjected the mice to dynamic PET imaging with tracer uptake under ketamine/xylazine anesthesia, which is described to lower ^18^F-FDG brain uptake (26). In this work, animals were awake during tracer uptake and subjected to static PET imaging. In fact, a study in healthy rats revealed that dynamic PET imaging with tracer uptake under different anesthesia protocols resulted in decreased ^18^F-FDG brain uptake compared to static PET imaging with awake tracer uptake (27). Thus, discrepancies might be due to differences in the PET protocol. On the other hand, metformin treatment in transgenic Tau-VLW mice, an animal model of tauopathies, elevated ^18^F-FDG uptake in the brain at 15 months of age (28), a similar effect as seen here in younger animals.

Similar to preclinical results, patients showed an increase in ^18^F-FDG uptake in hippocampus, parahippocampal gyrus and white matter regions and hypometabolism in frontal regions. A previous study has also reported increased uptake in cingulate and hippocampal regions, although not significantly, in cognitively impaired non-diabetic patients treated with metformin (29). These changes pair with the results shown here. In addition, Huang et al. have also reported increased uptake in white matter regions possibly due to inflammation in this area (16). In our animal study, we found a correlation between TSPO expression and hypermetabolism in a cortical subregion, which could support this hypothesis. However, given that accumulating evidence implies that metformin reduces neuroinflammation (17), we speculate an increase in the mitochondrial area in cytoplasm and the increase of mitochondrial metabolism transcripts (24) are a more likely explanation for this observation. The previous study also showed hypometabolic regions in the frontal lobe. However, the parahippocampal gyrus in these patients also showed decreased uptake in contrast to our findings. Thus, our data does not support their hypothesis that metformin is associated with a declining trend in memory performance that is driving the uptake reduction (16). This discrepancy could be due to differences in the study design. The control group in the study by Huang et al. consisted of diabetic patients whereas our control group consisted of healthy individuals. However, diabetes has been repeatedly associated with cerebral hypometabolism (30). In addition, the study performed by Luchsinger et al. with non-diabetic patients treated with metformin show also increased uptake and cognitive improvement (29). Therefore, it is not a likely explanation for the contradictory results of the two studies. Additionally, the studies were performed in different ethnic populations, i.e., in Taiwanese and Spanish individuals, respectively, which could potentially be associated with different responses to metformin treatment. Independently of the causes, the alteration of ^18^F-FDG brain uptake seen in this study in hippocampal areas, both in mice and patients, is of interest for PET imaging in neurodegenerative diseases. The hippocampus is one of the key regions affected in Alzheimer's disease, where hypometabolism in medial temporal areas is a recognized diagnostic hallmark (31). Therefore, metformin-induced hypermetabolism in this region could potentially mask pathological patterns, complicating the differential diagnosis among neurodegenerative diseases.

This study has some limitations. First, this is a retrospective study derived from a control population. All the patients in the metformin group are diagnosed with diabetes type 2 while the control group are healthy individuals. Thus, we have no reference group with diabetic patients without metformin treatment. Indeed, we found differences in glycosylated hemoglobin between both groups, being elevated in metformin-treated patients. Thus, differences could be driven by diabetes and not the treatment. However, hyperglycemia, insulin resistance, and diabetes have been found to be associated with cerebral glucose hypometabolism rather than hypermetabolism (30). With respect to the specific relationship between diabetes and cerebral glucose metabolism, a previous study evaluated brain ^18^F-FDG uptake in identical twins discordant in diabetes. They found hypometabolism in frontal, parietal, temporal and occipital lobes of diabetic individuals (32). Other studies comparing cerebral ^18^F-FDG uptake between diabetic and control patients also reported reduced uptake in diabetes (33, 34). Similarly, a mouse model of diabetes shows decreased ^18^F-FDG uptake in the brain (35). Indeed, we found a negative correlation between HbA1c and ^18^F-FDG uptake in frontal and parietal cortical regions but no correlation to hypermetabolic areas. Therefore, reduction in frontal areas in our patients might be driven by diabetes. However, hypermetabolism in white matter and temporal regions should be an effect of metformin treatment. Second, the sample size in both mouse and patient groups is small due to the exploratory and retrospective nature of this study. In addition to the small sample size, another limitation of the animal experiments is that we only used male mice due to the probing and complementary nature of these experiments. Furthermore, the dose used in this study represents a supraphysiological dose and this should be considered when extrapolating the preclinical findings to the clinical setting. Finally, FWE correction was not performed in SPM due to the small sample size of the groups. However, the clusters obtained support the results seen with the ROI analyses, suggesting the solidity of the parametric mapping, these results should be considered exploratory and interpreted with caution. Taking these limitations into account, further studies in larger and better controlled cohorts are warranted to validate our findings.

In conclusion, preclinical and clinical results show that metformin treatment is associated with regional alterations in ^18^F-FDG uptake, which may need to be considered in clinical interpretation. Therefore, nuclear medicine clinicians should be aware of these potential alterations when evaluating brain ^18^F-FDG PET.

## Data Availability

The data that support the findings of this study are available on request from the corresponding author. The data is not publicly available due to privacy or ethical restrictions.

## Supplementary material

SUPPLEMENTARY FIGURE S1 ROI templates used for analysis. Brain slices showing ROI distribution in (A) mouse and (B) human templates used for PET quantification. Legend shows areas' anatomical names and corresponding color codes.
