# Supplementary material for: Effect of metformin treatment on brain 18F-FDG uptake: a translational study
Source: Front Radiol. 2026 Oct 1;6:1917175. doi: 10.3389/fradi.2026.1917175 (PMC13669182; doi:10.3389/fradi.2026.1917175)
Supplement: Supplementary file 1 [file Table1.docx]

**Table S1. ^18^F-FDG uptake quantification in control and metformin-treated patients.** Table shows ROI SUVR value shown as mean±SD in all the brain regions evaluated. Right column shows p value of the t-test comparison between both groups. P-values below 0.05 (significant) are shown in bold.

| Region | Control | Metformin | p-value |
| --- | --- | --- | --- |
| Left Precentral Gyrus | 1.326±0.049 | 1.29±0.084 | 0.125 |
| Right Precentral Gyrus | 1.305±0.054 | 1.257±0.068 | **0.032** |
| Left Rolandic Operculum | 1.303±0.047 | 1.293±0.038 | 0.529 |
| Right Rolandic Operculum | 1.314±0.04 | 1.306±0.036 | 0.549 |
| Left Supplementary Motor Area | 1.37±0.052 | 1.302±0.058 | **0.001** |
| Right Supplementary Motor Area | 1.382±0.067 | 1.278±0.077 | **0.000** |
| Left Olfactory Cortex | 1.114±0.054 | 1.158±0.05 | **0.025** |
| Right Olfactory Cortex | 1.111±0.048 | 1.134±0.077 | 0.286 |
| Left Frontal Superior Gyrus | 1.293±0.062 | 1.243±0.044 | **0.019** |
| Right Frontal Superior Gyrus | 1.29±0.059 | 1.231±0.043 | **0.004** |
| Left Frontal Middle Gyrus | 1.377±0.064 | 1.33±0.057 | **0.043** |
| Right Frontal Middle Gyrus | 1.342±0.061 | 1.286±0.059 | **0.015** |
| Left Frontal Inferior Gyrus | 1.312±0.056 | 1.284±0.043 | 0.137 |
| Right Frontal Inferior Gyrus | 1.297±0.045 | 1.254±0.047 | **0.012** |
| Left Rectus Gyrus | 1.337±0.062 | 1.326±0.056 | 0.608 |
| Right Rectus Gyrus | 1.34±0.062 | 1.318±0.066 | 0.337 |
| Left Insula | 1.221±0.033 | 1.222±0.044 | 0.915 |
| Right Insula | 1.224±0.039 | 1.216±0.042 | 0.598 |
| Left Anterior Cingulate Gyrus | 1.203±0.063 | 1.206±0.063 | 0.884 |
| Right Anterior Cingulate Gyrus | 1.216±0.066 | 1.222±0.073 | 0.814 |
| Left Mid Cingulate Gyrus | 1.3±0.061 | 1.296±0.047 | 0.836 |
| Right Mid Cingulate Gyrus | 1.33±0.081 | 1.311±0.044 | 0.454 |
| Left Posterior Cingulate Gyrus | 1.423±0.084 | 1.391±0.093 | 0.312 |
| Right Posterior Cingulate Gyrus | 1.277±0.086 | 1.293±0.106 | 0.638 |
| Left Hippocampus Parahippocampus | 0.969±0.038 | 1.048±0.051 | **<0.001** |
| Right hippocampus Parahippocampus | 0.955±0.042 | 1.019±0.04 | **<0.001** |
| Left Amygdala | 0.855±0.059 | 0.946±0.064 | **<0.001** |
| Right Amygdala | 0.856±0.063 | 0.94±0.072 | **0.001** |
| Left Calcarine Sulcus | 1.472±0.066 | 1.442±0.062 | 0.211 |
| Right Calcarine Sulcus | 1.45±0.069 | 1.392±0.087 | **0.039** |
| Left Cuneus | 1.513±0.068 | 1.479±0.058 | 0.155 |
| Right Cuneus | 1.506±0.058 | 1.438±0.072 | **0.005** |
| Left Lingual Gyrus | 1.353±0.051 | 1.352±0.047 | 0.971 |
| Right Lingual Gyrus | 1.3±0.063 | 1.287±0.065 | 0.551 |
| Left Occipital Lobe | 1.314±0.043 | 1.308±0.049 | 0.721 |
| Right Occipital Lobe | 1.327±0.04 | 1.296±0.071 | 0.113 |
| Left Fusiform Gyrus | 1.229±0.05 | 1.29±0.039 | **0.001** |
| Right Fusiform Gyrus | 1.209±0.055 | 1.238±0.053 | 0.147 |
| Left Postcentral Gyrus | 1.272±0.051 | 1.243±0.071 | 0.165 |
| Right Postcentral Gyrus | 1.284±0.051 | 1.244±0.058 | **0.042** |
| Left Supramarginal Gyrus | 1.271±0.041 | 1.27±0.053 | 0.958 |
| Right Supramarginal Gyrus | 1.21±0.028 | 1.197±0.041 | 0.292 |
| Left Angular Gyrus | 1.324±0.084 | 1.275±0.064 | 0.090 |
| Right Angular Gyrus | 1.366±0.075 | 1.281±0.07 | **0.003** |
| Left Precuneus | 1.35±0.053 | 1.301±0.04 | **0.009** |
| Right Precuneus | 1.368±0.062 | 1.321±0.061 | **0.038** |
| Left Paracentral Lobule | 1.348±0.085 | 1.285±0.069 | **0.037** |
| Right Paracentral Lobule | 1.365±0.088 | 1.286±0.083 | **0.017** |
| Left Caudate | 1.374±0.091 | 1.375±0.137 | 0.991 |
| Right Caudate | 1.357±0.096 | 1.35±0.123 | 0.856 |
| Left Putamen | 1.511±0.091 | 1.483±0.075 | 0.377 |
| Right Putamen | 1.518±0.085 | 1.48±0.077 | 0.206 |
| Left Globus Pallidus | 0.978±0.049 | 0.975±0.049 | 0.867 |
| Right Globus Pallidus | 0.986±0.052 | 0.997±0.055 | 0.560 |
| Left Thalamus | 1.331±0.068 | 1.325±0.116 | 0.831 |
| Right Thalamus | 1.333±0.069 | 1.3±0.095 | 0.257 |
| Left Heschl Gyrus | 1.38±0.132 | 1.334±0.114 | 0.318 |
| Right Heschl Gyrus | 1.369±0.124 | 1.331±0.138 | 0.419 |
| Left Parietal Lobe | 1.328±0.066 | 1.276±0.067 | **0.038** |
| Right Parietal Lobe | 1.309±0.07 | 1.237±0.076 | **0.009** |
| Left Lateral Temporal Lobe | 1.191±0.027 | 1.197±0.031 | 0.538 |
| Right Lateral Temporal Lobe | 1.192±0.021 | 1.179±0.03 | 0.124 |
| Vermis | 1.303±0.124 | 1.346±0.092 | 0.302 |
| Left Cerebellum | 1.229±0.089 | 1.257±0.059 | 0.337 |
| Right Cerebellum | 1.188±0.082 | 1.227±0.062 | 0.167 |
| Midbrain | 1.138±0.059 | 1.177±0.071 | 0.092 |
